# Interaction of flexural-gravity waves in ice cover with vertical walls

**DOI:** 10.1098/rsta.2017.0347

**Published:** 2018-08-20

**Authors:** A. A. Korobkin, S. Malenica, T. Khabakhpasheva

**Affiliations:** 1 School of Mathematics, University of East Anglia, Norwich, UK; 2 Bureau Veritas, Marine and Offshore Division—Research Department, Paris, France; 3 Lavrentyev Institute of Hydrodynamics, Novosibirsk, Russia

**Keywords:** waves, elastic plate, rigid boundary

## Abstract

Diffraction of flexural-gravity waves in an ice cover by a bottom mounted structure with vertical walls is studied. The problem is solved by using the so-called vertical modes corresponding to the roots of the dispersion relation for flexural-gravity waves. These modes reduce the original three-dimensional problem to a set of two-dimensional diffraction problems with non-homogeneous boundary conditions on the rigid walls. Two unknown functions presenting in the boundary conditions for each mode are determined using the conditions at the contact line between the ice cover and the vertical walls. The clamped conditions at the contact line, where the ice cover is frozen to the wall, are considered in this study. The solution of the problem is obtained for a single vertical circular cylinder frozen in the ice cover. A general approach to the problem for vertical cylinders of any shapes is presented. The diffraction problems with vertical walls extended to infinity are discussed.

This article is part of the theme issue ‘Modelling of sea-ice phenomena’.

## 1. Introduction

**Figure 1.**
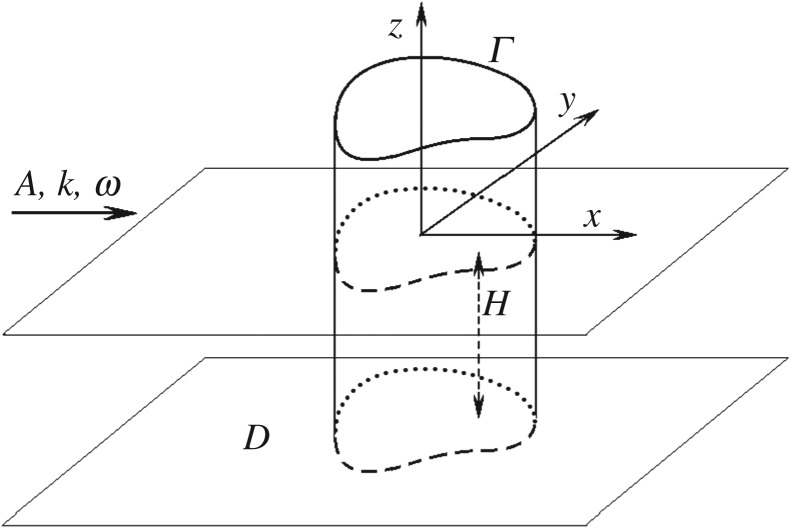
Sketch of the problem and main notations.

The linear three-dimensional problem of uni-directional flexural-gravity wave propagating in an infinite ice cover towards a vertical cylinder of an arbitrary cross-section *Γ* in water of finite depth *H* (figure 1) is studied by the vertical mode method in this paper. In two-dimensional problems of flexural-gravity waves reflected from a vertical wall this method was used in [1]. Other methods to study both two- and three-dimensional problems of hydroelastic waves and their interactions with vertical structures were developed in [2–8].

It was noted in [9] than the eigenfunctions, which represent the vertical modes of the liquid flow between the flat sea bottom and a floating elastic ice sheet, are non-orthogonal in a standard sense and could be incomplete. The two-dimensional scattering problem for a crack in the ice cover was solved by a Green function approach (see [9], §3) and then by the eigenfunction expansion method (see [9], §4), which is equivalent to the present method of vertical modes. It was reported in [9] that the vertical mode method is much simpler to use that the Green function method. The vertical mode method also gives useful details of the ice deflection and the flow beneath the ice. However, to validate the vertical-mode solution in [9], they solved the same problem by another method and demonstrated that these two solutions are identical. We followed the same idea and proved that the solutions of the linear diffraction problem for a vertical circular cylinder frozen in the ice cover of infinite extent by the vertical mode method and by the method based on the Weber integral transform in the radial coordinate, see [3], are identical. In this study, we generalize the method of vertical modes to any shapes of vertical walls. However, the numerical results are still shown only for circular cylinders. The vertical modes of ice sheet of constant thickness floating on water of finite depth were introduced in [10,11] and were successfully applied to two-dimensional problems of hydroelasticity without vertical boundaries in several papers.

This study is motivated by the need for some offshore structures, such as oil production platforms and wind farms, to be built far north in ice-covered waters [4]. The aim of the paper is to explain the application of the vertical mode method to three-dimensional problems of flexural-gravity waves interacting with vertical cylinders. If the wave-structure interaction problem has a unique solution, then the vertical mode method provides this solution, despite the fact that the vertical modes are linearly dependent and not necessary form a complete set of functions, see §5.

## 2. Formulation of the problem

The fluid flow and ice deflection are caused by an incident hydroelastic wave,
2.1
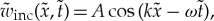
propagating in the positive *x*-direction (figure 1), where *A* is the amplitude of the incident wave, *k* is the wavenumber and *ω* is the wave frequency. A tilde stands for dimensional variables. Here real and positive *ω* and *k* are related by the dispersion equation of flexural-gravity waves. The linear problem of the incident uni-directional flexural-gravity wave interacting with a vertical bottom-mounted cylinder is formulated in non-dimensional variables (without tilde). The water depth *H* is taken as the length scale, 1/*ω* as the time scale, *A* is the scale of the deflections and *AHω* is the scale of the velocity potential of the flow. The ice deflection, *w*(*x*, *y*, *t*), and the velocity potential, *ϕ*(*x*, *y*, *z*, *t*), are periodic in time,
2.2
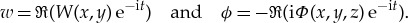
The complex potential, *Φ*(*x*, *y*, *z*), in (2.2) satisfies Laplace's equation,
2.3
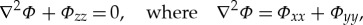
in the flow region, −1 < *z* < 0, (*x*, *y*)∈*D*. The plane *z* = − 1 corresponds to the flat rigid bottom, and the plane *z* = 0 corresponds to the ice–fluid interface. The potential *Φ* also satisfies the following boundary conditions,
2.4
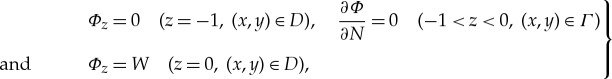
where ***N*** is the normal unit vector to the vertical wall *Γ* and ∂*Φ*/∂*N* is the normal derivative of the potential on the wall. The equation of thin ice plate can be written in the form, see [12],
2.5
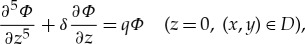
where *q* = (*ω*^2^*H*/*g*)(*H*/*L*_c_)^4^, *δ* = (1 − *ω*^2^/*ω*^2^_0_)(*H*/*L*_c_)^4^ and *L*_c_ = (*D*_*i*_/*ρg*)^1/4^ is the characteristic length of the ice sheet [13], *ω*_0_ = (*ρg*/*m*)^1/2^ is the frequency of floating broken ice, *m* is the mass of the ice cover per unit area, *m* = *ρ*_*i*_*h*_*i*_, *h*_*i*_ is the ice thickness, *ρ*_*i*_ is the ice density, *D*_*i*_ is the rigidity coefficient of the ice sheet, *D*_*i*_ = *E*_*i*_*h*^3^_*i*_/[12(1 − *ν*^2^)] for an elastic plate of constant thickness, *E*_*i*_ is the Young modulus of the ice, *ν* is the Poisson ratio, *ρ* is the water density and *g* is the gravitational acceleration. The condition at infinity follows from (2.1) and (2.2),
2.6
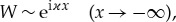
where ϰ = *kH* is the non-dimensional wavenumber. Condition (2.6) is imposed for *x*^2^ + *y*^2^ → ∞ if the vertical walls *Γ* do not extend to infinity. The three dimensionless parameters, *δ*, *q* and ϰ, are related by the dispersion relation [13],
2.7
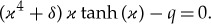
The conditions at the contact line, *z* = 0 and (*x*, *y*)∈*Γ*, between the ice cover and the surface of the cylinder can be complicated in practical problems. The present method of vertical modes is not sensitive to the types of these conditions. The method is demonstrated here for the ice cover being frozen to the vertical cylinder, which is modelled by the clamped conditions,
2.8
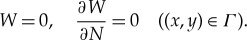


## 3. Vertical mode method

The solution of the formulated problem (2.3)–(2.7) is obtained by the method of separating variables. Within this method, a product *Φ*(*x*, *y*, *z*) = *W*_*n*_(*x*, *y*)*f*_*n*_(*z*) satisfies Laplace's equation (2.3) and the boundary conditions on the bottom (2.4) and the ice–water interface (2.5), if *f*_*n*_(*z*) is a non-trivial solution of the following spectral problem:
3.1
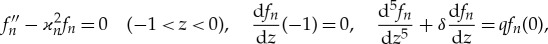
where ϰ_*n*_ is a root of the dispersion relation (2.7), *n* = − 2,  − 1, 0, 1, …, ϰ_0_ = ϰ and *W*_*n*_(*x*, *y*) is a solution of the equation
3.2
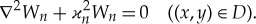
Note that, if ϰ is a root of (2.7), then −ϰ, 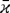 and 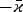 are also roots of this equation. Here a bar stands for complex conjugate of a complex number. In (3.1) and (3.2), we count only the roots of (2.7) with non-negative imaginary parts. Only such roots provide different vertical modes. Equation (2.7) has two real roots ϰ_0_ and −ϰ_0_, where ϰ_0_ > 0, infinite number of pure imaginary roots, ϰ_*n*_ = i*μ*_*n*_ and −ϰ_*n*_, where *n*≥1 and *μ*_*n*+1_≥*μ*_*n*_ > 0, and four complex roots, ϰ_−2_ = − *a* + i*b*, ϰ_−1_ = *a* + i*b*, −ϰ_−2_ and −ϰ_−1_, where *a* > 0 and *b* > 0 [9].

The solutions of the spectral problem (3.1), *f*_*n*_(*z*) = cosh[ϰ_*n*_(*z* + 1)]/(ϰ_*n*_sinh[ϰ_*n*_]), normalized by the condition *f*′_*n*_(0) = 1, are known as the vertical modes. They are orthogonal, 〈*f*_*j*_, *f*_*n*_〉 = 0, 〈*f*_*n*_, *f*_*n*_〉 = *Q*_*n*_, where *j*≠*n* and the scalar product of two functions *F*(*z*) and *G*(*z*), which are bounded together with their derivatives up to the third order in the interval −1 ≤ *z* ≤ 0, is defined by
3.3
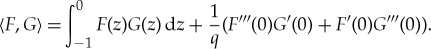
By algebra, *Q*_*n*_ = (ϰ^2^_*n*_(ϰ^4^_*n*_ + *δ*)^2^ + *q*(5ϰ^4^_*n*_ + *δ* − *q*))/(2ϰ^2^_*n*_*q*^2^). For the imaginary roots of the dispersion relation, ϰ_*n*_ = i*μ*_*n*_, where *μ*_*n*_ > 0 and *n*≥1, we have *μ*_*n*_ = *πn* − *q*(*πn*)^−5^ + *O*(*n*^−6^) as *n* → ∞. Therefore, *Q*_*n*_ = *O*(*n*^8^) as *n* → ∞. The conditions at infinity for equation (3.2), *n*≥ − 2, *n*≠0, correspond to outgoing waves diffracted from the vertical walls,
3.4
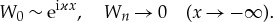
Then the solution of the original problem is given by the series,
3.5
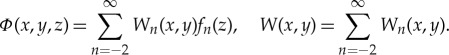


The boundary conditions on *Γ* for equation (3.2) are derived below in the local coordinates (*S*, *N*), where *N* = 0 on the vertical wall *Γ* and *S* is a curvilinear coordinate along the wall. Using (3.5_1_) we can calculate the derivative ∂*Φ*/∂*N* near the wall, where *N* > 0, by differentiating the series (3.5_1_) term by term. Then, to evaluate this normal derivative on the wall, we take the limit as *N* → 0. In this way, we satisfy the wall boundary condition (2.4_2_) as the limit where the boundary *Γ* is approached from the flow region *D*. This procedure of satisfying the boundary condition (2.4_2_) at the vertical wall *Γ* is needed here because the vertical mode method requires the derivatives up to the third order, see the definition of the scalar product (3.3). However, these derivatives are not continuous at the contact line. In particular, the second derivative ∂[(∂*Φ*/∂*N*)]/∂*z* is equal to zero on the vertical wall, (*x*, *y*)∈*Γ*, −1 < *z* < 0, including the limit as *z* → 0. On the other hand, the derivative ∂[(∂*Φ*/∂*z*)(*x*, *y*, 0)]/∂*N* = ∂*W*/∂*N*, is equal to zero as *N* → 0 only for the clamped conditions at the contact line. The boundary condition on the vertical wall (2.4_2_) is understood here as the limit where *N* → 0. In addition, the first and third derivatives in *z* at *z* = 0 in the definition of the scalar product (3.3) are understood as the limits of the derivatives *F*′′′(*z*), *G*′′′(*z*), *F*′(*z*) and *G*′(*z*) where *z* → 0^−^.

The boundary conditions on *Γ* for equation (3.2) are obtained by projecting (2.4_2_) on the vertical modes *f*_*k*_(*z*), *k*≥ − 2. By definition,
3.6
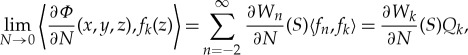
where (∂*W*_*k*_/∂*N*)(*S*) is the normal derivative of the function *W*_*k*_(*x*, *y*) on *Γ*. On the other hand, by using (3.3), the same limit can be calculated as
3.7
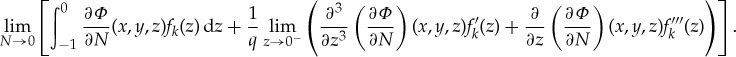


Here the limit of the integral is zero, see (2.4_2_), and *f*′_*k*_(0) = 1, *f*′′′_*k*_(0) = ϰ^2^_*k*_, see (3.1) and the normalization condition for the vertical modes. In the flow region, where *N* > 0 and −1 < *z* < 0, the potential *Φ* is continuous together with its derivatives. Then

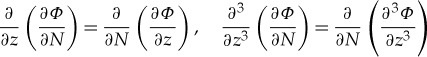
in the flow region, where equation (2.3) gives ∂^3^*Φ*/∂*z*^3^ = − ∇^2^(∂*Φ*/∂*z*). Taking the limit as *z* → 0^−^ in these equations and using the kinematic condition (2.4_3_), we find
3.8
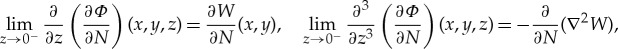
for *N* > 0. Finally taking the limit in (3.7) as *N* → 0 and equating the results of (3.6) and (3.7), we obtain
3.9
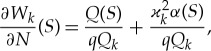
where *α*(*S*) = (∂*W*/∂*N*)(*S*) is the slope of the ice plate at the vertical wall *Γ* and *Q*(*S*) = − (∂/∂*N*)(∇^2^*W*) is the non-dimensional shear force on *Γ* with the scale *D*_*i*_*AH*^−3^.

Equation (3.9), *k*≥ − 2, provides the required boundary conditions at *Γ* for equation (3.2) in the flow region *D*. In these boundary conditions, the functions *Q*(*S*) and *α*(*S*) are unknown in advance and should be determined by using the conditions at the contact line. Note that these functions do not depend on the index of the vertical mode *k*, see (3.9).

The boundary conditions (3.9) show that the solutions *W*_*n*_(*x*, *y*) can be decomposed as
3.10
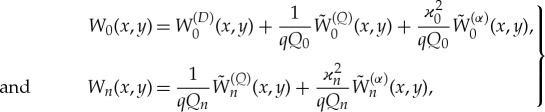
where *n*≥ − 2 and *n*≠0. The diffraction potential, *W*^(*D*)^_0_(*x*, *y*), is the solution of equation (3.2) with *n* = 0 such that 
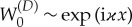
 as *x* →  − ∞, and (∂*W*^(*D*)^_0_/∂*N*)(*S*) = 0 on *Γ*. The radiation potentials 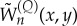 and 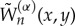, where *n*≥ − 2, are the solutions of equation (3.2), which describe the out-going waves at infinity and satisfy the following boundary conditions on the wall *Γ*,
3.11
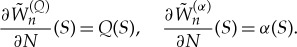


Equations (3.5), (3.10) and the boundary condition (3.11) provide the solution of the diffraction–radiation problem with the incident wave (2.1) and the following conditions at the contact line between the ice plate and the vertical wall *Γ*: both the slope and shear force of the ice plate at the vertical wall are given functions along the contact line,
3.12
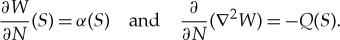
In the original variables, the ice plate slope and the shear force at the plate edge are also periodic functions of time with frequency equal to the frequency *ω* of the incident wave (2.1).

There are two difficulties with the derived solution. First, it should be explained how the solution with the edge conditions (3.12) can be used to solve the problems with other edge conditions, see §4. Second, it should be proved that the functions defined by (3.5), (3.10) and (3.11) indeed satisfy equations (2.3), (2.4) and (2.5). This will be done in §5 assuming that the solution of the problem is unique.

## 4. Vertical mode method for different edge conditions

The edge conditions (3.12) are artificial. Practical edge conditions for a thin elastic plate are the clamped conditions (2.8), simply supported conditions,
4.1
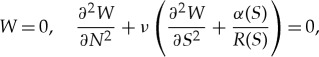
free–free conditions [14],
4.2
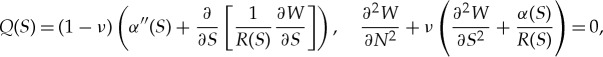
or mixed edge conditions on the contact line *Γ* between the vertical wall and the ice plate. Notations (3.12) are used in (4.1) and (4.2), and *R*(*S*) is the radius of curvature of the curve *Γ*. The boundary conditions (3.12) with *α*(*S*) = 0 and *Q*(*S*) = 0 are known as conditions of sliding edge, where the plate edge is free to move vertically but its rotation is not allowed [15]. For the sliding edge conditions, the displacement of the ice cover is given by (3.5), (3.10) and (3.11) as
4.3
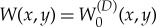
and can be determined in the same way as for water waves interacting with a vertical cylinder, see [16], but with the dispersion relation of the flexural-gravity waves (2.7).

### (a) General approach

To solve a flexural-gravity wave problem with specified edge conditions, a general solution of the problem with conditions (3.12) can be used, where the functions *α*(*S*) and *Q*(*S*) are unknown in advance now and should be determined as part of the solution. For example, for the clamped edge conditions (2.8), we should set *α*(*S*) = 0 and determine *Q*(*S*) such that the solution *W*(*S*) is zero on the plate edge. In general, actual edge conditions lead to a system of integral and differential equations for *α*(*S*) and *Q*(*S*). To derive these equations, we need the relations between the normal derivatives (3.11) on *Γ* and the solutions of (3.2) on *Γ*.

Equation (3.2) predicts that its solutions decay at any point of the flow region *D* as *n* → ∞, where ϰ_*n*_ = i*μ*_*n*_ and *μ*_*n*_ = *πn* + *O*(*n*^−5^), see §3. To find the asymptotic behaviours of the radiation potentials on the boundary *Γ* as *n* → ∞, we consider below the boundary problem for 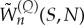. The problem for 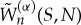 is considered in a similar way with *Q* changed to *α*. To satisfy the non-zero boundary conditions (3.11) for large *n*, we introduce the inner variables denoted by hats, 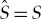 and 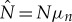, where 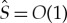 and 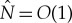 as *n* → ∞, and the new unknown function 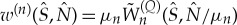. The function 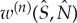 provides the solution close to the wall, where it satisfies the following equations
4.4
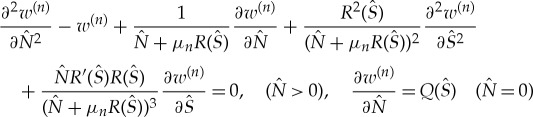
and decays at infinity, 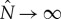. The asymptotic solution of the problem (4.4) reads
4.5
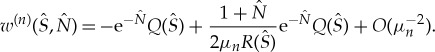
Then the values of the radiation potentials on the boundary *Γ* for large *n* are given by
4.6
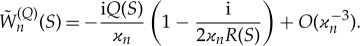
This asymptotic formula is valid also for 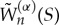 with *Q* changed to *α* in (4.6).

The required relations between the normal derivatives (3.11) on *Γ* and the solutions of (3.2) on *Γ* can be written now in terms of an integral non-local operator acting on *Γ*:
4.7
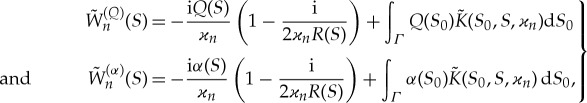
where the function 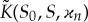 depends on the shape of the wall *Γ* and the coefficient ϰ_*n*_ in the field equation (3.2), where *k*≥ − 2. The asymptotic formula (4.6) provides that 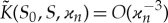 as |ϰ_*n*_| → ∞.

Substituting (3.10), (3.11) and (4.7) in (3.5), we find the solution *W*(*S*) on *Γ*:
4.8
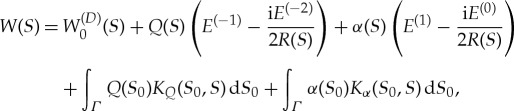
where

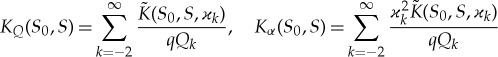
and

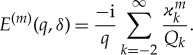
Once the functions *K*_*Q*_(*S*_0_, *S*) and *K*_*α*_(*S*_0_, *S*) are calculated for given shape of the vertical wall, then equations (3.12) and (4.8) can be used to determine *Q*(*S*) and *α*(*S*) for prescribed conditions at the contact line between the ice cover and the wall.

The free–free edge conditions (4.2) imply that both the effective transverse force (4.2_1_) and the bending moment (4.2_2_) are zero at the plate edge *Γ*. By using equations (3.12) and the Laplacian on *Γ* written in the local coordinates, ∇^2^*W* = ∂^2^*W*/∂*N*^2^ + (1/*R*)(∂*W*/∂*N*) + ∂^2^*W*/∂*S*^2^, condition (4.2_2_) is convenient to present in the form
4.9
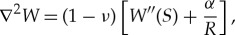
where
4.10
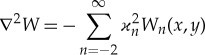
from equations (3.5) and (3.2). Equations (4.2) and (4.7)–(4.10) lead to the system of two integro-differential equations with respect to *Q*(*S*) and *α*(*S*). The system can be solved numerically.

For the clamped conditions (2.8), we have *α*(*S*) = 0 and equation (4.8) leads to the following integral equation for the function *Q*(*S*):
4.11
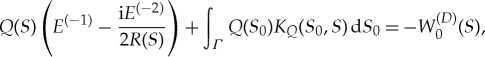
which is suggested to solve numerically. The main difficulty with the use of the above equation (4.11) is with the function *K*_*Q*_(*S*_0_, *S*) which can be determined by using the boundary integral equations for (3.2).

### (b) Fourier method for clamped edge conditions

The problem for a vertical cylinder frozen in an ice cover with clamped edge conditions can be also solved by using a complete set of functions orthonormal along the plate edge *Γ*, *g*_*m*_(*S*), where *m*≥0,
4.12
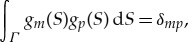

*δ*_*mp*_ is the Kronecker delta, and *g*_0_(*S*) = |*Γ*|^−1/2^ with |*Γ*| being the length of the plate edge. For the clamped edge conditions, we have *α*(*S*) = 0 and the function *Q*(*S*) is sought in the form of the series
4.13
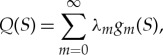
with unknown coefficients λ_*m*_. The functions 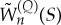 are the solutions of problem (3.2) and (3.11) on the boundary *Γ*. They can be obtained in the form
4.14
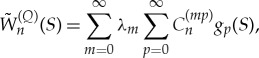
where the coefficients *C*^*mp*^_*n*_ are numerically determined by using the boundary integral equation of problem (3.2), (3.11). Here *C*^*mp*^_*n*_ = *C*^*pm*^_*n*_. Equations (3.5), (3.10) and (4.13) lead to the following symmetric system of algebraic equations for the coefficients λ_*m*_, where *p*≥0,
4.15
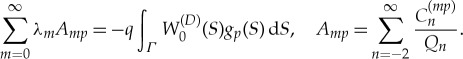
The right-hand side of equation (4.15) also can be evaluated by using the functions *g*_*m*_(*S*). The function *W*^(*D*)^_0_(*x*, *y*) is the solution of equation (3.2) with *n* = 0 such that 
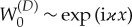
 as *x* →  − ∞, and (∂*W*^(*D*)^_0_/∂*N*)(*S*) = 0 on *Γ*. Let the edge of the ice plate *Γ* be described in the parametric form by the equations *x* = *x*_*Γ*_(*S*) and *y* = *y*_*Γ*_(*S*). The components of the unit normal vector to the ice edge are ***N***(*S*) = (*N*_*x*_(*S*), *N*_*y*_(*S*)). Then 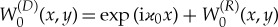, where the new unknown function *W*^(*R*)^_0_(*x*, *y*) satisfies (3.2) with *n* = 0, describes the outgoing waves at infinity, and satisfies the following boundary condition on *Γ*:
4.16
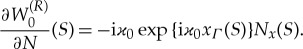
By introducing integrals
4.17
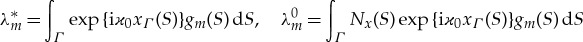
and using equation (4.14), we find the right-hand side in (4.15) in the form
4.18
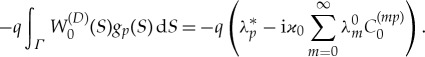
Therefore, the problem for clamped edge conditions can be reduced to calculations of the integrals λ*_m_, λ^0^_*m*_ and the coefficients *C*^(*mp*)^_*n*_ which are dependent on the shape of the vertical cylinder and the roots of the dispersion relation (2.7), where *m*, *p*≥0 and *n*≥ − 2. A similar approach with an expansion of the unknown function *α*(*S*) with respect to the orthonormal functions *g*_*m*_(*S*), see (4.13), can be used for other edge conditions.

The total vertical force *F*^(*V* )^_tot_(*t*) = ℜ(*F*^(*V* )^ e^−i*t*^) acting on the vertical cylinder is given by
4.19
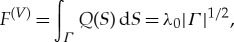
see equations (4.12) and (4.13). This force is caused by the shear force *Q*(*S*) along the edge of the ice plate. The scale of the vertical force is *D*_*i*_*A*/*H*^2^.

The total horizontal force ***F***^(*H*)^_tot_(*t*) = − ℜ(***F***^(*H*)^ e^−i*t*^),
4.20
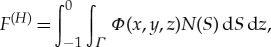
with the scale *ρAH*^3^*ω*^2^, can be written by using (3.5) and (3.10) as
4.21
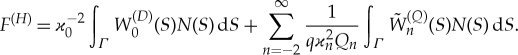
The integrals in (4.21) can be evaluated using the integrals similar to (4.17) and the coefficients of (4.14).

### (c) Fourier method for ice plate clamped to circular cylinder

For a circular cylinder of radius *B* in the non-dimensional variables and *S* being the curvilinear coordinate, *S* = *θB*, where *θ* is the polar angle, 0 ≤ *θ* < 2*π*, *x* = *r*cos*θ*, *y* = sin*θ*, *r* > *B*, we have |*Γ*| = 2*πB*, *g*_0_(*S*) = (2*πB*)^−1/2^, *g*_*m*_(*S*) = (*πB*)^−1/2^cos(*mθ*). The calculations provide *C*^(*mp*)^_*n*_ = 0 for *m*≠*p* and
4.22
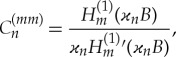
where *H*^(1)^_*m*_(*r*) is the Hankel function of the first kind corresponding to outward-propagating cylindrical waves. The diffraction potential *W*^(*D*)^_0_(*S*) on the cylinder is given by the MacCamy and Fuchs solution [17],
4.23
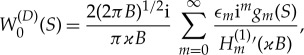
where *ϵ*_0_ = 1 and 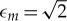 for *m*≥1. Substituting (4.22) and (4.23) in (4.15), we find the coefficients
4.24
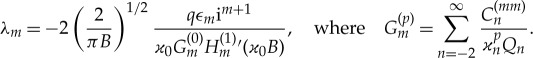
Equations (4.19) and (4.24) provide the formula for the vertical force acting on a circular cylinder frozen in an ice cover,
4.25
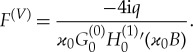
The component of the horizontal force (4.21) perpendicular to the direction of the incident wave, *F*^(*H*)^_*y*_, is equal to zero due to the symmetry of the problem. The force component in the direction of the incident wave is given by
4.26
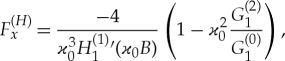


where the equation *N*_*x*_(*S*) = (*πB*)^1/2^*g*_1_(*S*) has been used. For long incident wave with ϰ_0_≪1, the force (4.26) approaches the force acting on a circular cylinder in water waves without the ice cover, see [4] for more details,
4.27
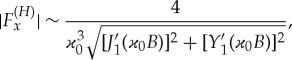
as ϰ_0_ → 0, which coincides with equation (4.10) in [4] and equation (7) in [17] after corresponding rescaling.

The strain distribution around the cylinder in incident waves is important for investigating the possibility for the ice cover to be broken due to the wave-structure interaction. The yield strain for the ice is estimated as 8 × 10^−5^ [4]. On the contact line of the cylinder frozen in ice, only the radial strain component, *ϵ*_*r*_(*S*, *t*) = (*Ah*_*i*_/2*H*^2^)*w*_*rr*_(*B*, *S*, *t*), is not equal to zero,
4.28
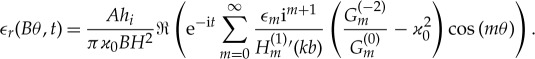
The hydroelastic behaviour of the ice cover around a circular cylinder in an incident linear wave is investigated numerically for the sea ice with density *ρ*_*i*_ = 917 kg m^−3^, Young 's modulus *E* = 4.2 × 10^9^ N m^−2^, Poisson's ratio *ν* = 0.33 and thickness 1.5 m. The mass of the ice sheet per unit area is *m* = 1375.5 kg m^−2^. The water density is *ρ* = 1026 kg m^−3^ and the water depth is *H* = 15 m. The characteristic length of this ice plate is *L*_c_ = 19.05 m, and the frequency of broken ice is *ω*_0_ = 2.7 s^−1^.

**Figure 2.**
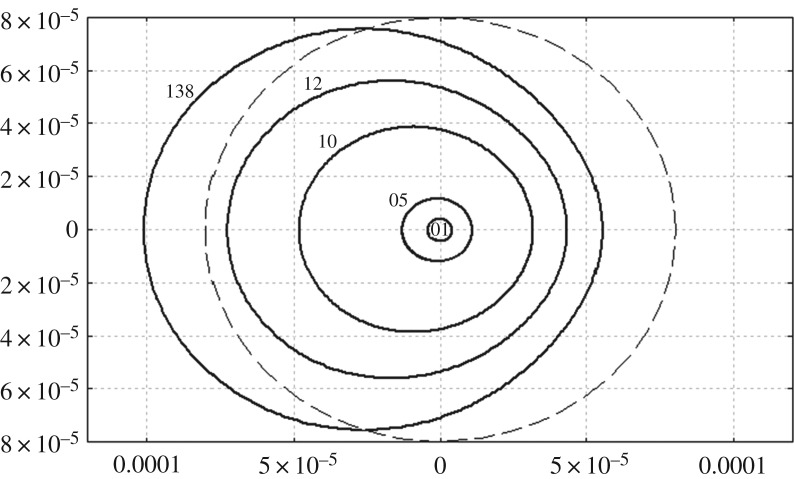
The radial strain as a function of the polar angle at the edge of the ice cover clamped to the circular vertical cylinder for ϰ_0_ = 0.1, 0.5, 1.0, 1.2, 1.38 and incident wave of amplitude 1 cm. The dashed circle corresponds to the yield strain 8 × 10^−5^. The incident wave propagates from left to right. The strains are smaller on the leeward side of the cylinder.

The amplitude of the radial strain as a function of the polar angle *θ* = *S*/*B* is shown in figure 2 for a circular cylinder of radius *b* = 5 m, wave amplitude of *A* = 1 cm and different wave length λ. The wave length with respect to the radius of the cylinder, λ/*b* is equal to 60*π*, 12*π*, 6*π*, 5*π* and 4.3*π* for the lines in figure 2, which gives roughly wave length of 942, 188, 94, 78 and 67 m. It is seen that the incident wave of amplitude 1 cm and length 67 m can break the connection between the ice plate and the cylinder along a part of the contact line. Note that the strains depend linearly on the wave amplitude *A*. Therefore, the wave of 94 m length, ϰ_0_ = 1.0, can damage the ice contact with the cylinder if the wave amplitude is 2 cm.

## 5. The solution by the vertical mode method

To prove that series (3.5), where *W*_*n*_(*x*, *y*) are given by (3.10), (3.2) and (3.11), and the functions *Q*(*S*) and *α*(*S*) are determined as explained in §4, provide the solution of the original problem (2.3)–(2.7) and satisfy the corresponding edge conditions on the contact line *Γ*, we assume that the solution is unique. Then we need to demonstrate that (i) the series (3.5) and their derivatives participating in the original equations converge and (ii) the equations of the problem are satisfied by the series.

The asymptotic formulae *Q*_*n*_ = *O*(*n*^8^), ϰ_*n*_ = *O*(*n*) and (4.6) as *n* → ∞ together with equations (3.10) and (3.2) yield *W*_*n*_ = *O*(*n*^−7^) and ∇^2^*W*_*n*_ = *O*(*n*^−5^). If *α*(*S*) = 0, then *W*_*n*_ = *O*(*n*^−9^) and ∇^2^*W*_*n*_ = *O*(*n*^−7^). In the following, *α*≠0. The vertical modes *f*_*n*_(*z*) for large *n*, where ϰ_*n*_ = i*μ*_*n*_ and *μ*_*n*_ = *πn* − *q*(*πn*)^−5^ + *O*(*n*^−6^), have the form
5.1
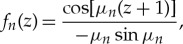
where *μ*_*n*_sin*μ*_*n*_ = *O*(*n*^−4^) as *n* → ∞. Therefore, *f*_*n*_(*z*) = *O*(*n*^4^) and the series (3.5) for *α*≠0 converge as *O*(*n*^−3^) and *O*(*n*^−7^), respectively.

Differentiating the series for *Φ*(*x*, *y*, *z*) in (3.5) twice in *x*, *y* and *z*, and using (3.1) and (3.2), we conclude that the series for ∇^2^*Φ* and for *Φ*_*zz*_ in −1 < *z* < 0 converges conditionally as cos[*πn*(*z* + 1)]/*n* and the Laplace equation (2.3) is satisfied term by term. Differentiating the series for *Φ*(*x*, *y*, *z*) in (3.5) five times in *z* and setting *z* = 0, see condition (2.5), we find that the series for the first, second and the third terms in (2.5) converge as *O*(*n*^−3^), *O*(*n*^−7^) and *O*(*n*^−3^) correspondingly. Therefore, condition (2.5) is satisfied by the vertical mode solution (3.5). The boundary condition on the bottom (2.4_1_) and the kinematic condition (2.4_3_) on the ice–fluid interface are satisfied term by term. The far-field condition (2.6) is not related to the convergence of the series (3.5), see equation (3.10) for details. The edge conditions are satisfied by the approach described in §4, once the solution of the original problem (2.3)–(2.7) with the artificial conditions (3.12) has been obtained. The only condition we should yet to explain is the condition on the vertical wall (2.4_2_).

Substituting (3.5) and (3.9) in (2.4_2_) at *Γ*, we find
5.2
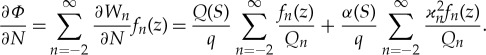
Condition (2.4_2_) is satisfied if both series on the right-hand side of (5.2) are equal to zero for any *z* from the interval [ − 1, 0]. Note that the values of the series depend on the roots of the dispersion relation (2.7) and the parameters *δ* and *q* in this equation. Both series converge absolutely. Equation (3.1) provides that the second series is equal to zero if the first one is zero for −1 < *z* < 0. In the first series, we have
5.3
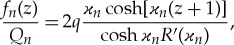
where *R*(ϰ) is the function on the left-hand side of the dispersion relation (2.7). It is convenient to introduce a function

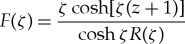
of complex variable *ζ* = *ξ* + i*η*, see [9] and a closed rectangular contour *C*_*M*_: *ζ* = *ξ* ± 2*πM*i, 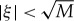 and 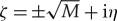, |*η*| < 2*πM*, where *M* is a positive integer. It is straightforward to show that the contour integral of *F*(*ζ*) over *C*_*M*_ tends to zero as *M* → ∞. On the other hand, the contour integral can be evaluated by the residue theorem, it is equal to the first series in (5.2) multiplied by 2*π*i/*q*. Therefore, the series is zero and the condition on the vertical wall is satisfied with the solution (3.5) for any functions *Q*(*S*) and *α*(*S*). This result indicates that the series (3.5) provide a solution of the original diffraction problem for flexural-gravity waves.

**Figure 3.**
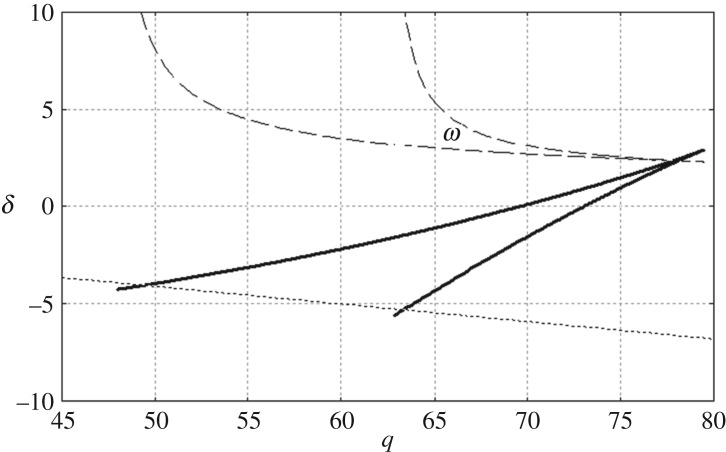
The pairs of the non-dimensional parameters (*δ*, *q*) providing double roots of the dispersion relation (2.7) are shown by the solid lines. The dashed lines show corresponding frequencies (in 1/s) of the incident wave for the ice characteristics of figure 2. The dotted line, *δ* = 0.3844 − 0.009*q*, relates the values of *δ* and *q* for these ice characteristics.

The result that both series in (5.2) are equal to zero for −1 ≤ *z* ≤ 0 indicates that the vertical modes, *f*_*n*_(*z*), *n*≥ − 2, are not independent and at least two of them can be presented as linear combinations of other functions, see [2]. Even the vertical modes are not independent, all of them are presented in solution (3.5). On the other hand, the set of the vertical modes could be not complete. We are unaware of a proof that the set *f*_*n*_(*z*), *n*≥ − 2 is complete on the interval −1 < *z* < 0. Moreover, each vertical mode corresponds to a certain root of the dispersion relation (2.7). For some parameters of the incident wave, it is possible that the dispersion relation has double and even triple roots. The forms of the vertical modes corresponding to double and triple roots are not clear yet. Such roots are pure imaginary. The values of the parameters *δ* and *q* providing double roots are obtained by solving the linear system *R*(ϰ, *δ*, *q*) = 0 and (∂*R*/∂ϰ)(ϰ, *δ*, *q*) = 0 with respect to *δ* and *q* for ϰ = i*μ*, *μ* > 0. The solutions of the linear system are shown in figure 3 by the solid lines. These lines are independent of any parameters. The values of *δ* and *q* at the cusp point provide a triple root. If the characteristics of the ice cover are given but the frequency of the incident wave varies, then the non-dimensional parameters *δ* and *q* are related by *δ* + *q*(*m*/*ρH*) = (*H*/*L*_c_)^4^. For the characteristics of the ice cover and the water depth from the calculations for figure 2 in §4, the latter line reads *δ* = 0.3844 − 0.009*q*, see dotted line in figure 3. The dimensional frequencies of the incident wave (in 1/s) corresponding to the double roots are shown by the dashed lines. It is seen that the calculated frequencies are in the range 2–5 s^−1^.

## 6. Conclusion

The method of vertical modes in the three-dimensional problems of flexural-gravity waves and their interaction with vertical walls has been described and applied to the problems with different conditions at the contact line between the ice cover and the vertical wall. The method can only be used for a constant water depth. In this study, the ice deflection is caused by an incident wave and the vertical wall represents a bottom-mounted vertical cylinder. It has been shown that the problem requires a general solution of an auxiliary problem with unknown in advance shear and deflection slope along the contact line. This general solution is obtained by using the vertical modes. Then the two unknown functions at the contact line are obtained using the physical conditions at the contact line. By using a set of orthogonal functions defined at the contact line, the latter problem can be reduced to a system of algebraic equations for any shape of the wall. This approach was applied to the problem of flexural-gravity wave interaction with a vertical circular cylinder frozen in the ice cover.

There are several open questions left after this study. It is not clear how to generalize the approach to problems with waves generated by a periodic external pressure near the walls. How can this method be used in problems with vertical walls extended to infinity? In particular, for an ice channel. It is not clear how to apply the method for conditions where the dispersion relation for the flexural-gravity waves has multiple roots. How can problems be solved with mixed conditions at the contact line? On the other hand, the method of vertical modes is very suitable for radiation problems with oscillating structures or parts of them.

## Data accessibility

This article has no additional data.
